# *Phragmites australis* management in the United States: 40 years of methods and outcomes

**DOI:** 10.1093/aobpla/plu001

**Published:** 2014-01-16

**Authors:** Eric L. G. Hazelton, Thomas J. Mozdzer, David M. Burdick, Karin M. Kettenring, Dennis F. Whigham

**Affiliations:** 1 Department of Watershed Sciences and Ecology Center, Utah State University, Logan, UT 84322, USA; 2 Smithsonian Environmental Research Center, 647 Contees Wharf Road, Edgewater, MD 21037, USA; 3 Biology Department, Bryn Mawr College, Bryn Mawr, PA 19010, USA; 4 Jackson Estuarine Laboratory, Department of Natural Resources and the Environment, University of New Hampshire, 85 Adams Point Road, Durham, NH 03824, USA

**Keywords:** Common reed, ecological restoration, herbicide, invasive plant, invasive species, management, *Phragmites australis*, watershed restoration.

## Abstract

Studies on invasive plant management are often short in duration and limited in the methods tested, and lack an adequate description of plant communities that replace the invader following removal. Here we present a comprehensive review of management studies on a single species, in an effort to elucidate future directions for research in invasive plant management. We reviewed the literature on *Phragmites* management in North America in an effort to synthesize our understanding of management efforts, identify gaps in knowledge and improve the efficacy of management. Additionally, we assessed recent ecological findings concerning *Phragmites* mechanisms of invasion and integrated these findings into our recommendations for more effective management. Our overall goal is to examine whether or not current management approaches can be improved and whether they promote reestablishment of native plant communities. We found: (i) little information on community-level recovery of vegetation following removal of *Phragmites*; and (ii) most management approaches focus on the removal of *Phragmites* from individual stands or groups of stands over a relatively small area. With a few exceptions, recovery studies did not monitor vegetation for substantial durations, thus limiting adequate evaluation of the recovery trajectory. We also found that none of the recovery studies were conducted in a landscape context, even though it is now well documented that land-use patterns on adjacent habitats influence the structure and function of wetlands, including the expansion of *Phragmites*. We suggest that *Phragmites* management needs to shift to watershed-scale efforts in coastal regions, or larger management units inland. In addition, management efforts should focus on restoring native plant communities, rather than simply eradicating *Phragmites* stands. Wetlands and watersheds should be prioritized to identify ecosystems that would benefit most from *Phragmites* management and those where the negative impact of management would be minimal.

## Abstract (precis)

We reviewed all available studies on *Phragmites australis* management in the United States. Our results show that there is a heavy emphasis on herbicides to manage *Phragmites*, relative to other methods, and a lack of information on what types of plant communities establish once *Phragmites* is removed. Our model of *Phragmites* establishment and reproduction describes the invasion as a symptom of watershed-scale land use and disturbance. We advocate more holistic approaches to control and management that focus on improving water quality and minimizing human disturbance to deter future invasion and improve resilience of native plant communities.

## Introduction

Wetlands are landscape sinks for nutrients and propagules, making them especially vulnerable to plant invasions as they are downstream from most disturbances (Zedler and Kercher 2004). One such invader, a Eurasian lineage of the common reed, *Phragmites australis* (hereafter referred to as *Phragmites*), is increasingly dominant in wetlands across North America (Marks *et al.* 1994; Chambers *et al.* 1999; Saltonstall 2003; Kettenring *et al.* 2012*b* in this issue). *Phragmites* invasions are often associated with decreases in plant biodiversity (Chambers *et al.* 1999; Keller 2000; Bertness *et al.* 2002), declines in habitat quality for fish and wildlife (Fell *et al.* 2003, 2006; Gratton and Denno 2006; Chambers *et al.* 2012), disruptions to biogeochemical cycles (Meyerson *et al.* 1999, 2000; Findlay *et al.* 2003) and other ecosystem services (but see Kiviat 2013 and Kettenring *et al.* 2012*b* in this special issue, which highlight *Phragmites* benefit to wildlife or lack/weaknesses of data on actual impacts). *Phragmites* invasion is becoming an increasingly large management concern in a variety of systems: tidal marshes along the Atlantic Coast (Chambers *et al.* 1999; Warren *et al.* 2001; Bertness *et al.* 2002); the Great Lakes (Tulbure *et al.* 2007; Carlson *et al.* 2009; Uzarski *et al.* 2009; Willcox 2013); inland brackish wetlands of the Great Basin (Kettenring and Mock 2012; Kettenring *et al.* 2012*a*) and the Gulf Coast (Kettenring *et al.* 2012*b* in this special issue).

*Phragmites* is a clonal, rhizomatous grass with a cosmopolitan distribution (Haslam 1972). Several genetic lineages, including some native lineages, are present in North America (Saltonstall 2002, 2003; Meyerson *et al.* 2012 in this special issue; Lambertini *et al.* 2012*a*, *b* in this special issue). However, the invasion by the Eurasian genetic lineage in wetlands across North America has been striking due to its rapid spread, abundance and impacts. Eurasian *Phragmites*' dominance at the landscape scale has been attributed to anthropogenic factors, including hydrologic alteration, increased nutrients and global change (Minchinton 2002*a*; Burdick and Konisky 2003; Silliman and Bertness 2004; Bart *et al.* 2006; King *et al.* 2007; Brisson *et al.* 2008; Mozdzer *et al.* 2010; Kettenring *et al.* 2011; Mozdzer and Megonigal 2012; Mozdzer *et al.* 2013 in this special issue). Since the turn of the 20th century, non-native *Phragmites* in North America has been associated with denuded soil and anthropogenic disturbance (Taylor 1938), but natural disturbances also produce favourable conditions for *Phragmites* establishment (Minchinton and Bertness 2003; Baldwin *et al.* 2010). *Phragmites* thrives in freshwater and brackish wetlands (Meyerson *et al.* 2000; Wilcox *et al.* 2003), and is expanding in managed systems like highway ditches (Lelong *et al.* 2007; Jodoin *et al.* 2008) and constructed wetlands (Havens *et al.* 2003).

*Phragmites* management strategies typically focus on the use of a limited number of techniques (described later) applied to individual patches or groups of patches. To critically and effectively evaluate restoration after an invasive species has been removed, data need to be collected to assess the initial wetland state, monitor the system through treatment (to inform adaptive management) and monitor for multiple years after treatment (see discussion in Blossey 1999). However, studies on the management of invasive plants (not just those investigating *Phragmites*) rarely report data beyond the response of the invader (reviewed in Reid *et al.* 2009), and monitoring for treatment effectiveness seldom lasts more than 2 years (reviewed in Kettenring and Reinhardt Adams 2011). A lack of long-term monitoring is likely due to: (i) the cultural mindset of land management agencies; and (ii) financial considerations and logistical constraints. *Phragmites* management in the USA has been occurring for over 35 years (Riemer 1976; Marks *et al.* 1994). Yet, while monitoring appears prohibitively expensive for specific projects, land managers spent over $4.6 million per year on *Phragmites* management across North America over a 5-year period (Martin and Blossey 2013), with no published data to justify the effectiveness of these management efforts to restore native plant communities. Given that eradication of *Phragmites* is rare, and is not likely without many years of follow-up treatments (Warren *et al.* 2002; Getsinger *et al.* 2006; Kettenring *et al.* 2012*a*; Lombard *et al.* 2012), monitoring of treatment effectiveness should be an essential component of any management programme.

Here we review current strategies for *Phragmites* management in North America and identify the factors that have the potential to transform future management. We begin with a literature review that addresses two central questions: (i) are current management practices successful? and (ii) do current *Phragmites* management practices allow for the restoration of native species assemblages? We address these questions by building upon earlier comprehensive reviews of *Phragmites* management (Marks *et al.* 1994; Kiviat 2006) in light of recent findings on the relationships among *Phragmites* invasion, land use and reproductive strategies within and among *Phragmites* patches. We also present a conceptual model of *Phragmites* invasion that integrates recent research findings. We argue that *Phragmites* management is best approached from a holistic perspective that integrates nutrient and disturbance management at landscape scales while addressing modes of reproduction and spread.

## Review of Existing Control Measures

### Methods

We reviewed the available literature on *Phragmites* management in the USA to determine: (i) which practices have been tested, (ii) where deficiencies in our knowledge exist, and (iii) what is known about recovery of native communities following attempts to eradicated *Phragmites*. We queried Google Scholar^®^ and ISI Web of Science^®^ for the technical and grey literature on *Phragmites* removal. We used the key words ‘*Phragmites* removal’ and ‘*Phragmites* management’ for all available dates. Articles, reports and theses from North America were included in our review (34 in total), along with reference to conclusions from previous reviews of the same topics. Only field studies that are applicable to management actions were included; meso- and microcosm studies are omitted. While our review focuses on non-native *Phragmites* in North America, they are presented in context with findings from other parts of the world. We did not consider *Phragmites* removal by hydrologic restoration in our quantitative review as that topic has recently been evaluated (Chambers *et al.* 2012); however, this approach is dealt with contextually when tied to another management method.

### Results and discussion

**Figure 1.**
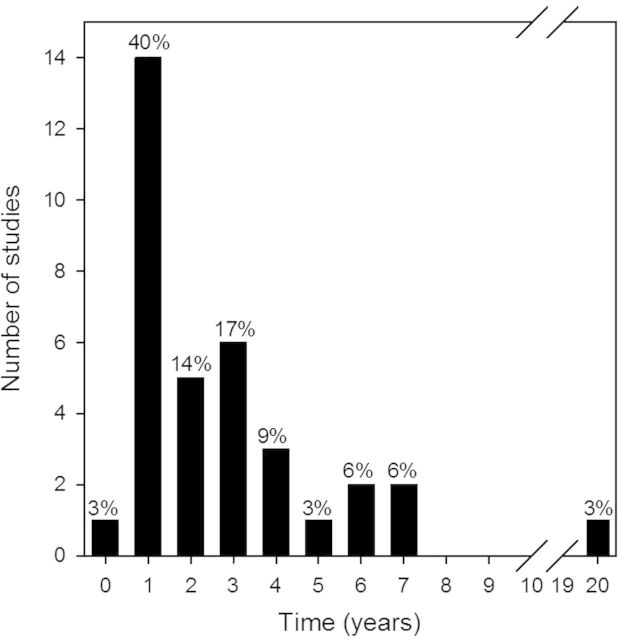
Duration of studies included in review. One study conducted a single survey and is denoted with the time = 0 bar.

**Figure 2.**
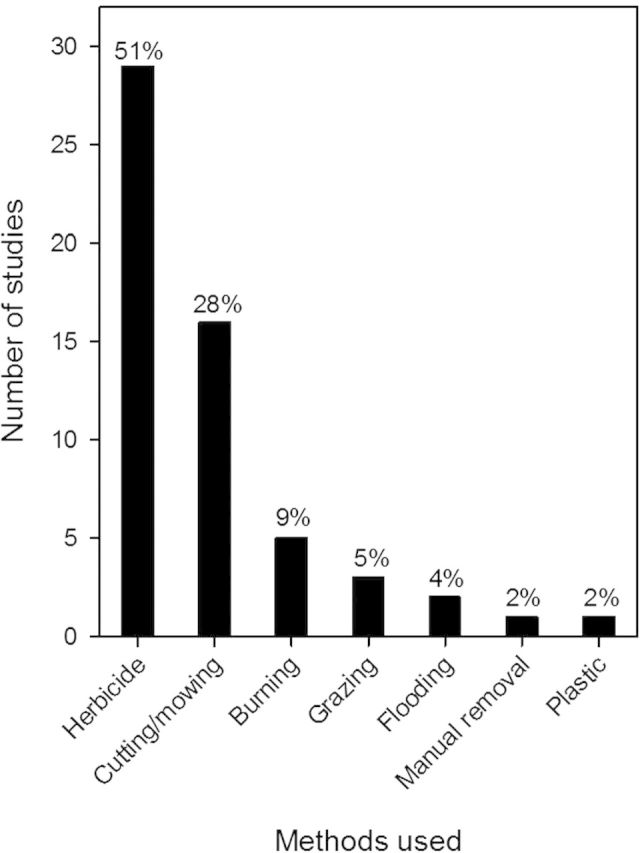
Management methods used in reviewed articles. Methods used in combination are counted individually.

**Figure 3.**
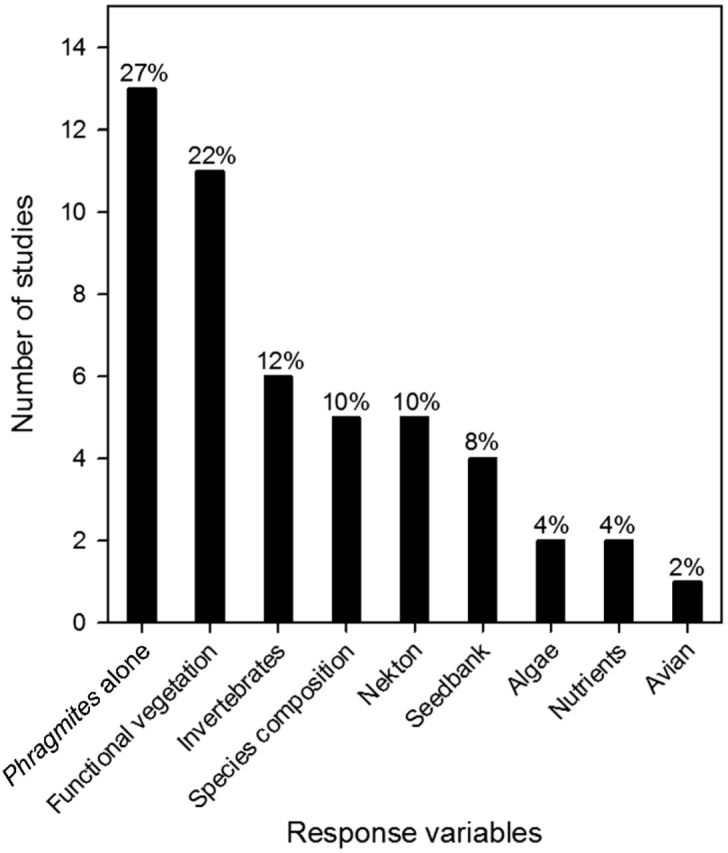
Response variables measured in reviewed studies. Functional vegetation represents only diversity, functional groups or species of interest, but not plant communities. Seedbank represents studies where germination trials were conducted.

The most common response variables measured in our review were *Phragmites*-only metrics or functional vegetation (vegetation type, diversity, etc.) (21/34 studies; Fig. 3). Several studies (5) quantified plant species composition following *Phragmites* management, although none performed any analysis that compared plant community composition. Additionally, only one study (Moore *et al.* 2012) compared recovering vegetation to reference sites, which is often critical in restoration and management (Neckles *et al.* 2002). Notably, two studies reported seed bank changes in response to *Phragmites* management and recorded ample seedbank for passive revegetation. Most studies (14) reported a single year of data and only 5 report >5 years of follow-up data, the most notable of which was a study that reported a 20-year follow-up observation (Fig. 1). The most commonly tested management technique was the use of herbicides (Fig. 2). Of the 34 studies, 27 reported results of the use of herbicides alone or in combination with other methods. A combination of cutting or mowing *Phragmites*, often in combination with flooding or herbicide use was studied in 15 instances (Fig. 2; Table 1).

**Table 1.** Studies included in quantitative review. Herbicide concentrations (rounded to 0.25 %) are reported for spray techniques alone and are reported as percent solution of commercial herbicide product in water. ^a^SB, seed bank composition; NU, nutrients; NK, nekton; AG, algae; IV, invertebrates; SC, species composition of nontarget plants; FV, functional vegetation (diversity, species of interest, native cover, etc.). ^b^‘G’ for glyphosate, ‘I’ for Imazapyr, ‘G + I’ for combined, ‘Varied’ if concentrations varied by site, ‘NR’ for studies that did not report concentrations, and ‘NA’ for studies that did not use herbicide. ^c^Indicates that study reported herbicide in mass of dry active ingredient, these values were converted to % solution based on the standard concentration of 58.3 % active ingredient in commercial herbicide blends (URS 2005). ^d^Combination of results from multiple studies.

Study	Location	Response variables^a^	Method	Duration (years)	Herbicide concentration^b^
Ailstock *et al.* (2001)	MD	SB, SC	Herbicide, mow, burn	4	G: 1.5 %
Back *et al.* (2012)	OH	PA, AG, IV	Herbicide	1	G: 30 %
					I: 5 %
Back and Holomuzki (2008)	OH	PA	Herbicide	7	G: 30 %
					I: 5 %
Brundage (2010)	MD	FV	Grazing (goats)	1	NA
Carlson *et al.* (2009)	Great Lakes	SB, SC, NU	Herbicide, cutting	2	G: NR
Derr (2008*a*, 12–16)	NJ	PA	Herbicide, mow	1	*G: 1.75 %
Derr (2008*b*, 153–157)	NJ	PA	Herbicide	1	G: 3 %
					I: 1 %
Farnsworth and Meyerson (1999)	CT	SC	Herbicide, mow	3	G: 1 %
Fell *et al.* (2003)	CT	FV, NK, IV	Herbicide, mow	1	G: 1.25 %
Fell *et al.* (2006)	CT	FV, IV, NK	Herbicide, mow	1	G: 1.25 %
Findlay *et al.* (2003)	CT	NU	Herbicide, mowing	3	G: 1 %
Getsinger *et al.* (2006)	MI	SC	Herbicide, burn, mow, flood	3/4	G: 3 %
					I: 1.5 %
					G + I: 2 % + 2 %
Gratton and Denno (2005)	NJ	IV	Herbicide	5	G: Varied
Hellings and Gallagher (1992)	DE	PA	Mow, flood	1	NA
Hallinger and Shisler (2009)	NJ	SB	Herbicide, cutting	5	G: 4 %
Kay (1995)	NC	PA	Herbicide (wipe on), Mow	2	NA
Kimball and Able (2007)	DE	NK	Herbicide, burn	1	G: Varied
Knezevic *et al.* (2013)	NE	PA	Herbicide	1	G: Varied
					I: Varied
					G + I: Varied
Kulesza *et al.* (2008)	OH	AG, NK, IV	Herbicide (wipe on)	2	NA
Lazaran *et al.* (2013)	OH	FV, AV	Herbicide	1	NR
Lombard *et al.* (2012)	MA	PA	Herbicide (clip and drip, wipe on, spray)	7	G: 2 % (spray)
Mozdzer *et al.* (2008)	VA	FV	Herbicide	1	G: 2 %
					I: 2, 5 %
Myers *et al.* (2009)	VA	SB, FV	Herbicide	6	I: 6 %
Myers *et al.* (2007)	VA	PA	Herbicide	4	I: 10 %
Plentovich (2008)	WI	FV	Herbicide, burn, mow	1	I: 2.5 %
URS (2005)	DE	FV	Grazing, mowing, herbicide (wipe on, spray) excavation	6	G: Varied
Rapp *et al.* (2012)	NE, WY	PA	Herbicide, mowing, disking	3	G: 4 %
					I: 4 %
Riemer (1976)	NJ	PA	Herbicide	3	^c^G: 2.25, 4.25, 6.5 %
Smith (2005)	MA	PA	Manual	1	NA
Tesauro and Ehrenfeld (2007)	NJ	FV	Grazing (cattle)	Single survey	NA
Turner and Warren (2003)^d^	CT	FV	Herbicide	20	^1^G: 1.25 %
					^1^G: Varied
Warren *et al.* (2001)	CT	NK, SC, IV	Herbicide, mow	2	G: 1.25 %
Wang *et al.* (2006)	NJ	FV	Herbicide, planting	3	NR
Willcox (2013)	CT	PA	Plastic	1	NA

Our review focused on four main categories of methods for controlling *Phragmites*: mechanical, chemical, biological and novel methods. Here we review these methods to discuss their effectiveness and to highlight research needs.

#### Mechanical control

Mechanical control is perhaps the first human reaction to remove an unwanted plant, and the methods vary in efficacy and degree of effort. It is largely achieved with mechanical mowing or cutting with hand tools, hand-pulling, crushing, excavation of entire plants, burning or cutting, often followed by covering the area with soil or plastic.

#### Mowing and cutting

For a perennial rhizomatous grass, mowing does little to reduce its dominance. Mowing actually stimulated shoot production and resulted in increased density of *Phragmites* shoots (but decreased shoot height and biomass) in both non-tidal (Güsewell *et al.* 1998; Güsewell 2003; Asaeda *et al.* 2006; Derr 2008*a*) and tidal wetlands (Warren *et al.* 2001).

Variable results following cutting were likely due to a combination of phenology, abiotic conditions and patch size. Impacts from cutting vary relative to the phenology of the plant, due to shoot/rhizome interactions, as reserves are mobilized and stored differently according to season (Weisner and Granéli 1989; Asaeda *et al.* 2006 and references therein). For example, cutting in June showed significant impacts to aboveground and rhizome biomass the following growing season, whereas cutting in July showed no significant impacts compared with controls (Asaeda *et al.* 2006) and open wetlands to pelagic flushing (Uzarski *et al.* 2009). External environmental factors (e.g. temperature and salinity) can influence success; cutting just before the flooding season has been reported to improve control (Marks *et al.* 1994; Kiviat 2006). Some researchers report cutting treatments are less effective when soils are sandy or aerated (Weisner and Granéli 1989). One primitive approach broke shoots and removed them by hand (several shoots were held tight and broken below the waterline as the bases were kicked) along shorelines of five fresh water ponds (Smith 2005). High water levels in all ponds resulted in broken/crushed shoots remaining underwater for an extended period and mortality ranged from 41 to 99 % after 1 year (Smith 2005).

On a large scale, hand cutting will largely be ineffective due to time and resources, but may be an important strategy of rapid response efforts. Overall, simply cutting will be ineffective in eliminating *Phragmites*, but with proper timing, cutting may help reduce dominance (through depletion of underground reserves) and control expansion.

The most effective means of *Phragmites* mechanical control is a combination of cutting or mowing (usually in the spring) and covering stubble with plastic (for one growing season). However, there are limitations to this application; it is usually applied to small areas, as it is labour-intensive (Dawson and Hallows 1983; Boone *et al.* 1988; Marks *et al.* 1994; Kiviat 2006; Willcox 2013). In one removal experiment, *Phragmites* shoot density averaged 0.1 m^−2^ beneath the plastic compared with 20.7 m^−2^ in plots without plastic (Burdick *et al.* 2010). Thus, unless cutting is combined with plastic sheeting or herbicide, mowing alone will have little effect on *Phragmites* management other than containment.

#### Burning

Burning of *Phragmites* provides an alternative mechanism for physical removal, similar to mowing, but burning has not been effective unless coupled with either hydrological restoration or herbicide application (Marks *et al.* 1994). Burning alone has produced variable results and even stimulated *Phragmites* growth and stand development (van der Toorn and Mook 1982; Thompson and Shay 1985; Cross and Fleming 1989; Granéli 1989).

Cutting and burning appear to enhance control efforts if used as secondary treatments. For example, mechanical control efforts improved significantly following either herbicide use (Carlson *et al.* 2009) or the reintroduction of flood waters in tidal wetlands (Hellings and Gallagher 1992; Teal and Peterson 2005; Getsinger *et al.* 2006). In some instances, burning to remove standing dead biomass in winter was found to enhance control following restoration of tidal exchange (Sun *et al.* 2007). Burning aboveground shoots (or other methods like cutting or crushing) followed by flooding can be used to cut off the oxygen flow to the rhizomes (Weisner and Granéli 1989; Rolletschek *et al.* 2000).

Removal or mulching of the aboveground material following cutting has been recommended (Marks *et al.* 1994; Kiviat 2006), even though removal and disposal involves more effort to prevent recolonization from rhizomes. Burning removes the dead thatch and aids in the regeneration of native plants (Ailstock *et al.* 2001)—typically a primary goal where managers wish to control *Phragmites*. Removal by either mechanism also increases light availability that warms exposed soils. Such conditions enhance germination and recruitment of native plants from seed banks, which is critical for wetland recovery (Marks *et al.* 1994; Farnsworth and Meyerson 1999; Ailstock *et al.* 2001; Kiviat 2006; Carlson *et al.* 2009).

#### Excavation

Excavation provides complete *Phragmites* control, and is likely the only landscape-scale option for mechanical removal, but requires disproportionally greater costs in both time and resources. Land managers have successfully restored *Phragmites*-dominated dredge spoil sites to highly valued salt marshes in New England (Moore *et al.* 2009). In such cases, excavation to elevations at or below mean high water (i.e. coupling removal with restoration of hydrology) results in daily tidal flooding, increased salinity and sulfide, and resulted in restoration of native plant communities and associated faunal species in Connecticut and New Hampshire (Moore *et al.* 2009).

### Chemical control

#### Herbicide

Herbicides are currently the primary tool used by land managers to control or eliminate *Phragmites* in North America (94 % in a recent national survey; Martin and Blossey 2013; and 97 % in Utah alone, Kettenring *et al.* 2012*a*). There are several application methods and two main herbicide active ingredients (glyphosate and imazapyr) that have been used with varying levels of success (see recent herbicide comparison by Cheshier *et al.* 2012). Perhaps one of the greatest challenges in understanding the efficacy of herbicides on *Phragmites* management is the lack of data on the long-term impacts of herbicide application on *Phragmites* and non-target species (Figs 1 and 3). In addition, few studies have specifically addressed different application rates and/or application time (Back and Holomuzki 2008; Derr 2008*b*; Mozdzer *et al.* 2008; Back *et al.* 2012; Cheshier *et al.* 2012). The majority of the data that we found were not reported in peer-reviewed publications but in technical reports and bulletins in the ‘grey literature’ which are rarely readily available. We divide information on the use of herbicides into (i) herbicide types and their effects on ecosystem recovery, and (ii) a comparison of herbicide efficacy and potential effects on non-target vegetation.

#### Glyphosate

The most commonly used herbicides contain the active ingredient glyphosate; this is likely attributed to the fact that glyphosate herbicides were the only Environmental Protection Agency (EPA)-approved herbicides for application in aquatic environments until 2003. Common trade names approved for aquatic application of glyphosate to control *Phragmites* include Rodeo™, GlyPro™ and Aqua Neat™. As a broad-spectrum systemic herbicide, glyphosate is non-selective and also kills non-target plants including woody and herbaceous plants. According to the Rodeo™ label, glyphosate is taken up through the plant epidermis and subsequently moves into the root system through the vascular tissue. In the plant, it interferes with amino acid synthesis specifically found in plants and microorganisms. Degradation of glyphosate is reported to occur through microbial pathways in <7 days; however, greenhouse studies have reported the persistence of glyphosate or glyphosate-related products for up to 79 days (Meyerson *et al.* 1997), suggesting that any subsequent replanting should occur several weeks after replanting dates given by the label instructions, due to potential negative effects on non-target native plants. A surfactant must be added to aid in foliar uptake, and reported toxicity in fauna has been attributed to surfactants in the various formulations (Tu *et al.* 2001), and not the herbicide itself.

Historically, glyphosate was applied at the end of the growing season (per label instructions) when plants were translocating resources to belowground rhizomes. Owing to the extremely long growing season of non-native *Phragmites* (Farnsworth and Meyerson 2003; League *et al.* 2006), it was possible to apply glyphosate after native plant senescence with minimal effect on native vegetation. Two recent studies have found that, contrary to label instructions, earlier application of glyphosate (June *vs.* September) is more effective at controlling *Phragmites* (Derr 2008*b*; Mozdzer *et al.* 2008). However, earlier application also has the potential to negatively impact native plants (Mozdzer *et al.* 2008), which is often at odds with management goals.

**Figure 4.**
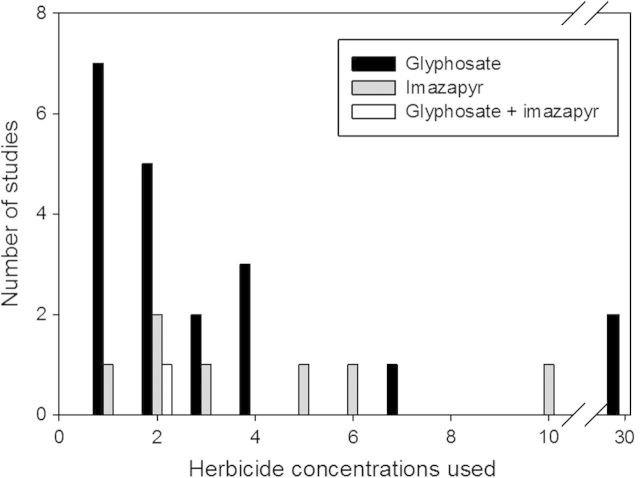
Herbicide concentrations (as percent solution of active ingredient in water) used by herbicide removal studies.

The use of glyphosate-containing herbicides usually requires multiple applications over successive years to be effective. Unfortunately, no published studies exist that have evaluated how many applications of glyphosate are necessary for complete *Phragmites* control. We speculate that the effectiveness of any herbicide is likely related to the amount of belowground reserves, abiotic conditions and applicator error. However, there is an urgent need to understand the appropriate control application methods to reduce excess herbicides from entering wetland systems (see concentrations tested in Fig. 4).

#### Imazapyr

The active ingredient imazapyr was approved in 2003 by the US EPA for application in wetland habitats labelled as Habitat™, Eagre™ and EcoImazapyr™. Since then, land managers have been using this herbicide (Marris 2005; Clarke 2006) to control *Phragmites*. According to the label, imazapyr works by a mechanism targeting broad-chained plant-specific amino acids in meristematic regions, and is translocated belowground to kill rhizomes. Unlike glyphosate, imazapyr is taken up by the plants’ leaves as well as by its roots. In solution, imazapyr is broken down through photodegradation with an average half-life of 2 days. However, in soils where ultraviolet breakdown does not occur, microbial breakdown of imazapyr is the primary mechanism of degradation with half-lives ranging from 1 month to over 4 years (Tu *et al.* 2001) with soil moisture, soil depth, pH and temperature affecting the rates of microbial degradation (Vizantinopoulos and Lolos 1994). Toxicity is described as low to birds and mammals; however, non-ionic surfactants may have detrimental effects on invertebrates (Tu *et al.* 2001).

Controlled comparative studies have found that imazapyr is more effective than glyphosate in controlling *Phragmites* (Kay 1995; Getsinger *et al.* 2006; Derr 2008*b*; Mozdzer *et al.* 2008), but not without serious negative consequences to native plants including recolonization following the death of *Phragmites* (Mozdzer *et al.* 2008). The only studies that reported glyphosate exhibiting a greater impact on *Phragmites* under field conditions were two that used higher concentrations than recommended by the manufacturer (30 % in study *vs.* <6 % recommended) and were not comparable to the rate of imazapyr used (5 %) (Back and Holomuzki 2008; Back *et al.* 2012) (Fig. 4). Other studies have demonstrated that there is no need to use glyphosate in concentrations higher than those listed on the product label (Cheshier *et al.* 2012), and label instructions should not be exceeded due to potential negative consequences on flora and fauna. Land managers have noted that wetlands are slower to recover when imazapyr is used when compared with glyphosate herbicides (Mozdzer *et al.* 2008), which may be attributed to greater persistence in the soil. Given the potential for non-selective root uptake of imazapyr by all plants, the presence of imazapyr or imazapyr residues may be affecting the seed banks of native plants. Research is critically needed to understand whether imazapyr has negative impacts on the seed bank, or if the delayed recovery can be attributed to the persistence of the herbicide in the soils impairing growth of seedlings.

#### Landscape-scale *Phragmites* control programmes using herbicides

Few have investigated or attempted to control *Phragmites* at the landscape level, and even fewer have made the results available to the scientific community. Perhaps one of the largest restoration projects occurred on the Delaware River as part of the Public Service Electric and Gas restoration. Several papers (Turner and Warren 2003; Gratton and Denno 2005; Teal and Peterson 2005; URS 2005; Kimball and Able 2007) were published midway through the restoration process, reporting on the management approach, but the final results assessing if the management objectives to restore vegetatively diverse, functioning wetlands were achieved have never been published as a peer reviewed study.

In Virginia, USA, land managers have established one of the most thorough management and coordination programmes that we are aware of by combining efforts with private, state and federal stakeholders (Myers *et al.* 2009). Partnering with numerous public and private entities, state staff targeted priority conservation areas (the coastal habitats of Virginia around Chesapeake Bay) to reduce the cover and rate of *Phragmites* spread. These efforts spanned 6 years and often included an initial aerial application that was followed by ground-based applications in subsequent years to control any re-sprouting. Most of the sites that were treated were surveyed by helicopters in 2004 and 2008. Given that the treatments and surveys were coordinated at the landscape level (Myers *et al.* 2009), the effort enabled land managers to share resources, resulting in one of the few examples of landscape-scale management and control.

The coordinated work in Virginia (Myers *et al.* 2009) revealed several patterns, which provided insights for future management. In treated areas, land managers were able to reduce *Phragmites* abundance by 34 % from 706 acres to 468 acres. However, where aerial control was not applied, there was a 22 % increase in *Phragmites* abundance from 657 to 805 acres. Cumulatively over a 4-year period, *Phragmites* abundance was only reduced by 4 % total since management focused primarily on large stands (>5 acres). However, during this same period, the small (<0.25 acres) and medium (>0.25 and <5.0 acres) sized class populations increased in abundance by 22 and 87 %, respectively, accounting for almost all the gains in habitat from controlling the large stands. These findings suggest that targeting large stands may not be appropriate for controlling *Phragmites* at the landscape level. Instead, priority should be given to small patches that are likely to expand in the future and may contribute to future expansion by sexual reproduction (Myers *et al.* 2009), which is an approach supported in general recommendations for invasive species control (Moody and Mack 1988).

Regardless of the herbicide used, one-time applications are never 100 % effective (Kettenring and Reinhardt Adams 2011). In order for a control and restoration programme to be successful, land managers must commit to multi-year applications (e.g. Riemer 1976; Kay 1995; Warren *et al.* 2001; Cheshier *et al.* 2012; Lombard *et al.* 2012) in addition to a long-term commitment from land managers and stakeholders (Teal and Peterson 2005).

### Biological control

#### Plant competition

Plant competition by native plants can alter the restoration trajectory. Unmanaged areas where *Phragmites* has been controlled effectively, but not replanted with native species, are often reinvaded by *Phragmites* immediately either by seeds or regrowth from rhizomes that were not killed. The importance of *Phragmites* seed banks in reinvasion varies. Earlier studies reported that *Phragmites* was not present in the seed bank (Van der Valk and Davis 1979; Wilson *et al.* 1993; Baldwin and DeRico 1999); however, more recent studies have found ample *Phragmites* seed in the seed bank (Smith and Kadlec 1983; Welling *et al.* 1988*a*, *b*; Leck 2003; Baldwin *et al.* 2010). As a grass, *Phragmites* seeds do not remain viable in the seed bank for very long. Where germination of *Phragmites* seeds has been reported, the density of the germinated seeds can be almost as high as the number of viable seeds produced (∼700 seeds m^−2^, Baldwin *et al.* 2010). If this scenario is typical, it suggests that revegetation of areas from which *Phragmites* has been killed should be planted or seeded with native plants as soon as possible, under the theory that native plants will competitively exclude *Phragmites* seedlings (Farnsworth and Meyerson 1999; Wang *et al.* 2006; Carlson *et al.* 2009; Byun *et al.* 2013). Field experiments in tidal marshes have shown that native plants, though smaller, can slow the recolonization of *Phragmites* seedlings (Minchinton 2002*b*; Minchinton and Bertness 2003) and reduce the success of resprouting from rhizomes (Amsberry *et al.* 2000; Konisky and Burdick 2004; Wang *et al.* 2006; Peter and Burdick 2010).

Greater species richness in resident plant communities may reduce the ability of *Phragmites* to colonize and expand. A wetland with intact vegetation will have fewer opportunities for *Phragmites* colonization (Kennedy *et al.* 2002). The potential of native species to successfully compete with *Phragmites* was demonstrated in a field experiment in which one or four native species were planted with *Phragmites* shoots that were grown from rhizomes. Plots with greater species richness had the most dramatic effects, reducing *Phragmites* shoot density >50 %, biomass >90 % and survival >65 % compared with unplanted controls (Peter and Burdick 2010). A Canadian competition study evaluated plant functional diversity as a factor in *Phragmites* competition. Byun *et al.* (2013) found that biotic resistance in plant communities increased by niche preemption (native plants germinated before *Phragmites* seeds) and niche partitioning (functional diversity). These two experiments demonstrate the importance of plant communities and post-control revegetation in resisting *Phragmites* invasion.

Accelerated development or succession provides an alternative management strategy. This strategy can be successful where the vegetation of forested wetlands or upland edges of wetlands has been disturbed and replaced by *Phragmites*. Here, removal could be coupled with planting trees and shrubs to shade out *Phragmites* (Kiviat 2006; Geoff Wilson, Northeast Wetland Restoration, pers. comm*.*). A survey of *Phragmites* invasion of 15 created tidal wetlands found *Phragmites* stands decreased cover where shrub/scrub habitat developed (Havens *et al.* 2003). This approach may prevent *Phragmites* reestablishment over the long term, or may allow only scattered *Phragmites* plants to survive.

Native seed banks are critical for successful revegetation after *Phragmites* removal. The literature is full of conflicting results, but overall, wetlands tend to have diverse persistent seed banks (Leck and Simpson 1995; Ungar 2001; Leck 2003; Leck and Leck 2005) and seed bank studies have not resulted in any clear relationship between the diversity of species in the seed bank and *Phragmites* invasion. In a Great Lakes study, Carlson *et al.* (2009) found that the diversity of vegetation after *Phragmites* removal depended upon the diversity of the native seed bank. It has also been shown that a diverse native seed bank can persist in monocultures of *Phragmites* (Baldwin *et al.* 2010). In fact, the diversity of herbaceous species in the seed bank has been found to be greater in stands dominated by *Phragmites* compared with surrounding areas dominated by native vegetation (Minchinton *et al.* 2006). Minchinton and colleagues concluded that the high cover of *Phragmites* and the thick litter layer inhibited the germination of non-*Phragmites* seeds in the seed bank. In a tidal freshwater system, Ailstock *et al.* (2001) found that the seed bank under *Phragmites* and after *Phragmites* removal both had a high diversity of species. These authors concluded that the type of *Phragmites* management will alter the seed bank, with herbicide-burn treatments having a different seed bank species composition compared with herbicide alone which impacts the outcome of passive revegetation. Hallinger and Shisler (2009) reported successful recolonization of native vegetation from the seed bank alone (with minor reseeding) in a New Jersey salt marsh following *Phragmites* removal. In New England, greater plant diversity was found in treated areas compared with both invaded and uninvaded controls (Moore *et al.* 2012). These studies indicate that the seed bank can play an important role in any wetland restoration effort following *Phragmites* removal.

#### Herbivory

Grazing has long been used to manage *Phragmites* stands, primarily in Europe (Marks *et al.* 1994), yet there are very few empirical studies evaluating grazing in North America (reviewed in Kiviat 2006). Tesauro and Ehrenfeld (2007) used grazing to manage *Phragmites* and other invasive species in a New Jersey wetland and found the method beneficial to plant species diversity and animal habitat, but the study lacked replication. Brundage (2010) showed that in Maryland, goats can significantly decrease *Phragmites* density, height and biomass while concurrently increasing species diversity in grazed plots. Around the Great Salt Lake in Utah, several agencies use grazing to manage *Phragmites*, primarily using cattle (49 % of surveyed land managers in Kettenring *et al.* 2012*a*). Although there are no formal monitoring data available, wetlands in Utah that receive high-intensity, short-duration grazing appear to respond best, with *Distichlis spicata* replacing *Phragmites* after 3 years of grazing rotation (Rich Hansen, Utah Department of Wildlife Resources, pers. comm.), and increases in shorebirds and waterfowl as well (Chad Cranney, Utah Department of Wildlife Resources, pers. comm.). In contrast, a study that tested goat grazing in New Jersey marshes in low densities (∼1 goat per acre) found that goats preferentially ate all vegetation except *Phragmites*, only consuming *Phragmites* when all other options were exhausted (Teal and Peterson 2005; John Teal, J. M. Teal Associates, pers. comm.; URS 2005). Forced grazing in small plots, where grazing mammals do not have an alternative food source, can be successful in controlling *Phragmites* if applied appropriately (B. R. Silliman *et al.*, in review). However, there are obvious tradeoffs associated with high-intensity grazing, such as soil compaction, trampling and/or nutrient enrichment that may prevent it from being a suitable method in many areas. Diverse communities of natural herbivores also help suppress *Phragmites* expansion. Small mammals appear to decrease establishment of *Phragmites* in lower salinity tidal marshes (Gedan *et al.* 2009). Muskrats graze *Phragmites* in freshwater systems in the western United States (E.L.G.H., pers. observ.) and brackish wetlands (T.J.M., pers. observ.), indicating that natural herbivory will influence species assemblages in wetlands that contain *Phragmites*. Natural grazing by small mammals may be fostered in brackish marshes by providing muskrat platforms and enhancing habitat for natural herbivores (see Kiviat 2006). Other natural herbivores seem deterred by *Phragmites* (*Litorina irrorata*; Hendricks *et al.* 2011). There is little information on how either natural herbivory or targeted grazing allow for the reassembly of native plant communities.

#### Classical biocontrol organisms

Biocontrol organisms are currently highly prioritized by land management agencies as a low-cost management strategy alternative. Traditional biocontrol agents are insect herbivores found in the invasive plant's native range that can have strong impacts on its growth and reproduction (Tscharntke 1999; Van Driesche *et al.* 2010). Planned introductions of invertebrates are often controversial as there is a potential for unintended effects on non-target organisms or even across trophic levels (Thomas and Reid 2007), with only 27 % of studies reporting complete success in eliminating invasive plants (Van Driesche *et al.* 2010). A recent survey of land managers found that 91 % would release biocontrol organisms for *Phragmites*, indicating that there is a strong desire for new techniques to control this grass (Martin and Blossey 2013). Some land managers expressly prohibit the use of biocontrols due to the potential for unintended impacts and the risks to non-target organisms (Tu *et al.* 2001). The search for a biocontrol for *Phragmites* in North America has been going on for over a decade (Tscharntke 1999; Tewksbury *et al.* 2002; Blossey 2003; Häfliger *et al.* 2005), and several potential insect biocontrols have been identified and are currently undergoing host-specificity testing with potential releases in 2–3 years from time of writing (B. Blossey, Cornell University, pers. comm.).

In the native range of Eurasian *Phragmites*, there are several dozen invertebrate herbivores in reed stands (Tscharntke 1999) and many of the natural enemies are also found in North America (see Tewksbury *et al.* 2002 for a comprehensive review). Indeed, *Phragmites* herbivores are still being discovered in North America (Eichiner *et al.* 2011). Several herbivores prefer native conspecific *Phragmites* to the non-native lineage (Lambert *et al.* 2007), findings that are troubling given the potential impacts on the widely distributed native *Phragmites* in North America. The herbivores currently present in North America are not considered effective at controlling the spread of the invasive form of *Phragmites*, though some can prevent flowering (e.g. *Lipara* spp*.*, Lambert *et al.* 2007). An ongoing study in the Chesapeake Bay has found stem infection rates by insects of over 50 % (E. L. G. Hazelton *et al.*, in review), yet the degree of impact on competitive dominance and reproductive output is yet to be studied.

### Novel methods in *Phragmites* management

Several new management methods are currently in development, ranging from hydrologic restoration to alteration of rhizosphere conditions, novel molecular tools and fungal pathogens. Multiple research groups are investigating pathogens as potential biocontrols. A group at Cornell University is looking at oomycetes as a potential *Phragmites* management tool (Nelson 2009). Shearer and Harms (2012) attempted to isolate fungal pathogens that will preferentially attack non-native *Phragmites* in North America. In a converse approach, another group is using fungal inhibitors to eliminate endophytes in *Phragmites* and then assess reductions in performance (USGS Great Lakes Science Center 2012, 2013). Gene silencing techniques are in development with a goal of identifying knock out genes associated with *Phragmites* growth and photosynthesis (USGS Great Lakes Science Center 2012).

In tidal wetlands, restoring hydrology often results in increased porewater sulfide shifting the competitive advantage to native vegetation over *Phragmites* (Warren *et al.* 2001; Chambers *et al.* 2003; Moore *et al.* 2012). High concentrations of sulfide impede nutrient uptake (Chambers *et al.* 1999) and also decrease *Phragmites* growth (Howes *et al.* 2005). Observations of lower sulfide levels in tidal marsh soils with *Phragmites* stands suggest that high sulfide levels may limit *Phragmites* distribution (Chambers *et al.* 1999, 2002). Seeds, seedlings and cuttings can tolerate sulfide concentrations of up to ∼1.5 mM sulfide (reviewed in Chambers *et al.* 2003), but mature culms were able to survive consistent sulfide levels of 1.5 mM (Howes *et al.* 2005). These findings suggest that mature stands with clonal connections may be tolerant of high sulfide concentrations. Therefore, hydrologic control might work best following mechanical actions to eliminate aboveground portions of mature shoots, preventing *Phragmites* from oxygenating the rhizosphere.

Other invasive grasses have been successfully managed by nitrogen control including *Bromus tectorum* (Kulmatiski and Beard 2006; Vasquez *et al.* 2008) and *Phalaris arundinacea* (Ianone *et al.* 2008). Vasquez *et al.* (2008) found that more holistic management practices consisting of controlled grazing, microbial change (through carbon amendment) and native planting helped control nitrogen and make sites less invasible by *B. tectorum* in semi-arid systems. In other systems, addition of sawdust to promote microbial nitrogen immobilization, combined with planting diverse plant assemblages allowed native species to recover following management for *P. arundinacea* (Ianone *et al.* 2008). Sawdust addition impacts non-native grasses more than non-native and native forbs and native grasses (Alpert and Maron 2000). Sugar amendment decreased the success of multiple invasive plants greater than adding activated charcoal (Mitchell and Bakker 2011). Even carbon amendment will likely require watershed-scale restoration to permanently decrease plant-available nitrogen (Perry *et al.* 2010) and future studies will need to determine the efficacy of such approaches on *Phragmites*.

Based on this review, we see the need for more research that investigates comprehensive, landscape-scale, integrative management strategies. There is a clear bias in the literature to herbicide use and mowing or cutting, which is reflected in recent surveys of land managers (Kettenring *et al.* 2012*a*; Martin and Blossey 2013). These methods may be effective on a site-by-site basis, but they do not address the factors that contribute to *Phragmites* invasion. Whether the management goal is to eliminate *Phragmites* or merely reduce its dominance, control measures will be more successful if linked with establishment of native plants to occupy the site and periodic monitoring to identify, mark and treat invasive plants. Regardless of the control method and initial success of native plants, non-native *Phragmites* will recolonize in most cases (unless salinities are high, as in Sun *et al.* 2007) and will be difficult to eliminate from invaded wetlands (Farnsworth and Meyerson 1999; Warren *et al.* 2001).

## Integrating Recent Insights about *Phragmites* Ecology into Management: A Conceptual Model

**Figure 5.**
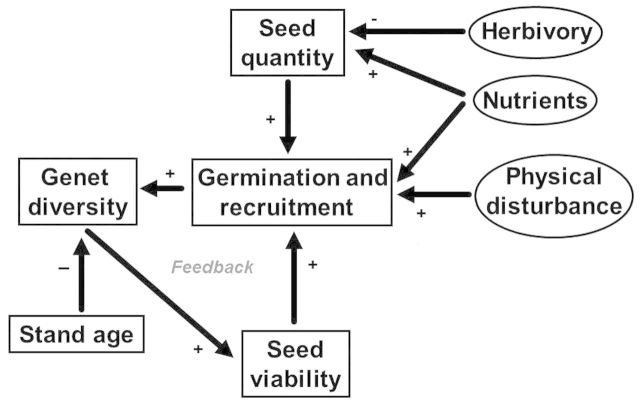
Conceptual model of *Phragmites* spread. Intrinsic factors are shown in boxes; extrinsic factors are in ovals. Genet diversity has a positive effect on viable seed production due to increased out-crossing potential. There is a positive feedback between the intrinsic factors affecting sexual reproduction and spread that are further enhanced by physical disturbances and nutrients.

Plant invasions, including that of *Phragmites*, are triggered by both intrinsic and extrinsic factors and are typically interactions between nutrients, disturbance and propagule pressure (Colautti *et al.* 2006). Intrinsic factors are aspects of a species' biology that drive its establishment and spread. Extrinsic factors include anthropogenic disturbances, nutrient enrichment and herbivory. We developed a conceptual model of *Phragmites* spread that is driven by interactions between intrinsic and extrinsic factors (Fig. 5). This model can be used to guide future efforts to manage *Phragmites.* The model is comprised of four intrinsic components that positively affect spread: (i) seed quantity; (ii) seed viability; (iii) germination and recruitment; and (iv) genet diversity. In our model, germination and recruitment are central to increasing genet diversity (outcrossing potential). Increased genet diversity through outcrossing potential leads to an increase in seed viability (Kettenring *et al.* 2010, 2011; McCormick *et al.* 2010*a*, *b*). Increases in seed quantity or seed viability will result in higher recruitment rates (new clonally diverse *Phragmites* stands), feeding the cycle. Stand age is an intrinsic factor that slows this feedback loop. Three extrinsic factors are nutrients, disturbances and herbivory; the first two of which positively impact spread while herbivory has a negative effect through reductions in seed production. Nutrients and physical disturbance also fuel the cycle by increasing seed quantity and recruitment (nutrients), and creating microsites for germination (disturbance). We describe each of these components in greater detail below.

*Phragmites* invasions were long thought to originate primarily from vegetative propagules (e.g. rhizomes) on the upland edge of wetlands (Bart *et al.* 2006), despite the fact that *Phragmites* is capable of sexual reproduction and spread from seed. Seed is dispersed by wind or birds (Haslam 1969; Soons 2006) and new molecular evidence has made it increasingly clear that seeds, rather than vegetative propagules, are the primary means of reproduction for colonization by *Phragmites* (Campbell 2007; Brisson *et al.* 2008; Baldwin *et al.* 2010; Belzile *et al.* 2010; Kettenring *et al.* 2010, 2011; McCormick *et al.* 2010*a*, *b*; Kirk *et al.* 2011; Kettenring and Mock 2012).

Viable seed production in *Phragmites* is driven by outcrossing potential, a phenomenon that is enhanced in polyclonal patches (Kettenring *et al.* 2010, 2011). Viable seeds will lead to the production of new clones, thereby increasing outcrossing potential in a positive feedback that is further enhanced by the presence of disturbances and nutrients (Kettenring *et al.* 2010, 2011; McCormick *et al.* 2010*a*, *b*). In particular, inflorescence size and seed quantity increase with elevated nutrients (Kettenring *et al.* 2011), and *Phragmites* in watersheds with a greater degree of anthropogenic development produce more seeds than those with less human impact (King *et al.* 2007; Kettenring and Whigham 2009; Baldwin *et al.* 2010; Kettenring *et al.* 2010; McCormick *et al.* 2010*a*). *Phragmites* seedlings then can exhibit ‘explosive growth’ in response to elevated nutrients (Saltonstall and Stevenson 2007).

*Phragmites* is a disturbance specialist and its seeds require light and large diurnal temperature fluctuation to break dormancy; conditions typically found on bare, non-inundated soils (Armstrong *et al.* 1999; Ekstam and Foresby 1999; Ekstam *et al.* 1999). Bare soils can be the result of anthropogenic or natural events such as burial by wrack (Minchinton 2002*a*; Minchinton and Bertness 2003), a water level drawdown (Smith and Kadlec 1983; Galinato and Van der Valk 1986; Welling *et al.* 1988*a*, *b*; Tulbure *et al.* 2007; Whyte *et al.* 2008; Tulbure and Johnston 2010; Wilcox 2012), or removal of litter and vegetation by wave action (Baldwin *et al.* 2010). Specific conditions for seed germination are found in the upper edge of wetlands where there is ample oxygen (Wijte and Gallagher 1996*a*) and salinities are typically low (Wijte and Gallagher 1996*a*, *b*; Greenwood and MacFarlane 2006). Then the plant expands primarily through vegetative means via rhizome or stolon extension (Amsberry *et al.* 2000; Bart *et al.* 2006). Although susceptible to flooding during early stages, seedling tolerance to flooding increases with age (Wijte and Gallagher 1996*b*; Mauchamp *et al.* 2001; Baldwin *et al.* 2010; also see review in Weisner and Granéli 1989; Clevering 1999; Engloner 2010).

Clonal diversity decreases with stand age (Koppitz *et al.* 1997; Koppitz and Kuhl 2000; Curn *et al.* 2007; Krivackova-Sucha *et al.* 2007), potentially decreasing future sexual reproduction by decreasing outcrossing potential. Thus, older stands may decrease in management priority as their clonal diversity decreases. Hyper-adapted clones will be able to prevent seeding establishment by shading the underlying substrate. The outcome of these interactions is that a single clone may eventually competitively exclude other clones, potentially decreasing future sexual reproduction by decreasing outcrossing potential. Many of the oldest stands in Chesapeake Bay appear to have decreased their rate of spread (Rice *et al.* 2000), perhaps as a wetland reaches carrying capacity.

In addition to stand age effects on sexual reproduction, several obligate *Phragmites* endophagous herbivores eliminate *Phragmites* apical dominance, thus destroying flowering potential on attacked culms (e.g. *Lipara* spp., *Giraudiella* spp., *Calamomyia* spp., *Lasioptera* spp., *Tetramesa* spp. in Tscharntke 1999; Tewksbury *et al.* 2002; Lambert *et al.* 2007). While the total impact of herbivory on seed production at the stand or population level is not clear, rates of attack can reach levels likely to decrease seed production substantially (often >50 % of stems attacked, Lambert *et al.* 2007; >90 % E. L. G. Hazelton *et al.*, in review).

Watershed-scale changes in land use resulting from development, and associated increases in disturbances and the availability of limiting nutrients such as nitrogen, contribute to *Phragmites* invasion (Bertness *et al.* 2002; Silliman and Bertness 2004; King *et al.* 2007). *Phragmites* presence is linked to development at or near the shoreline (Bertness *et al.* 2002; King *et al.* 2007). The absence or disruption of forested buffers at the upland–wetland–estuarine ecotone edge has been shown to result in expansion of *Phragmites* in New England (Burdick and Konisky 2003; Silliman and Bertness 2004) and the Chesapeake Bay (King *et al.* 2007; Chambers *et al.* 2008). Greater wave energy and watershed-scale nutrient loading interact to increase sexual reproduction and clonal diversity in *Phragmites* stands (Baldwin *et al.* 2010; Kettenring *et al.* 2011). Once wetlands within nutrient enriched watersheds have been invaded, *Phragmites* can spread rapidly through sexual reproduction and the subsequent dispersal of seeds (McCormick *et al.* 2010*a*; Kettenring *et al.* 2011). Anthropogenic vectors (highways and boat transport) promote the transport and expansion of *Phragmites* between watersheds and across the landscape (Lelong *et al.* 2007; Jodoin *et al.* 2008; Kettenring *et al.* 2012*a*, *b* in this special issue).

Our model of *Phragmites* spread and reproduction is consistent with observations in other species, where increasing nutrient availability and physical disturbance make ecosystems more susceptible to invasion (Alpert *et al.* 2000; Richardson and Pysek 2012). In order to truly manage *Phragmites*, we will need to work at the watershed scale to make sites less able to be invaded through nutrient management and decreased anthropogenic disturbance (Alpert *et al.* 2000) and create conditions that do not favour seed production. Nitrogen management may become the most effective means to control *Phragmites* in the future (Kettenring *et al.* 2011), especially with climate change and increasing CO_2_ (Mozdzer and Megonigal 2012). Efforts at the watershed scale to promote ‘restoration to ensure resilience’ (Suding 2011) are needed to combat spread from seed. In addition, addressing sexual reproduction as part of management efforts will be critical (Kettenring *et al.* 2011), especially given that the common practice to control *Phragmites* in the fall with glyphosate often occurs after seeds have been produced (Marks *et al.* 1994; Kettenring *et al.* 2011).

## Conclusions

Critiques of *Phragmites* management are not new, and some authors have called for revaluation of *Phragmites* and the tradeoffs associated with management. Several authors have demonstrated that non-native *Phragmites* provides valuable ecosystem services, especially in the context of increasing anthropogenic stressors and climate change. The services include providing resilient vegetation (Ludwig *et al.* 2003), accretion rates that can keep pace with sea level rise (Rooth *et al.* 2003), habitat quality (Meyerson *et al.* 2010), nutrient removal (Mozdzer *et al.* 2010) and other ecosystem services (Rooth and Windham 2000; Kiviat 2006, 2013 in this special issue; Hershner and Havens 2008). The potential ecosystem services provided by *Phragmites* must be weighed against the desired management outcomes (such as waterfowl management; Cross and Fleming 1989) associated with *Phragmites* removal. Since we still know little about the composition of vegetation communities after *Phragmites* is removed, we should weigh the costs of management heavily against the assumed benefits. *Phragmites* management has a great economic cost (Martin and Blossey 2013) and could be met with public backlash due to the use of herbicide and other cultural perceptions (Teal and Peterson 2005). It is unlikely that a single strategy will work at all sites; and all management actions should be conducted in a case-specific manner with considerations for the likelihood of success and the costs of management in each watershed.

Managers may decide that certain landscapes have been altered too far from a natural state to successfully control *Phragmites* and have reached an alternate stable state that includes non-native *Phragmites* monocultures. Choosing to restore sites that are less degraded and facilitating native plant communities are critical steps toward successful management of invasive plants (Reid *et al.* 2009). Research and land managers should focus on identifying and restoring sites that are likely to recover and remain *Phragmites* free (*sensu*: Ailstock *et al.* 2001; Reid *et al.* 2009). Restoration efforts may not succeed at all unless they are conducted at the watershed scale in order to address the initial cause (or source) of the invasion (Palmer 2009). Based on our model of *Phragmites* invasion, sites that are in low nutrient watersheds where physical anthropogenic disturbances are unlikely should resist invasion (also see discussion in Kettenring *et al.* 2010). Large-scale comparative studies that manage *Phragmites* across multiple watersheds will help us determine the factors that contribute to success and failure in *Phragmites* restoration efforts (*sensu* Suding 2011). Once established, *Phragmites* is difficult to remove; preventing invasion may be more efficient than control. *Phragmites* control programmes that focus on protection of non-invaded wetlands through prioritization will likely be more successful than those aiming to reduce or eliminate *Phragmites* in heavily invaded watersheds.

The actual outcomes of *Phragmites* removal are still largely unclear. In perhaps the most comprehensive study to date, Ailstock *et al.* (2001) recommended site-specific management with clearly defined restoration objectives. Restoration and management efforts that remove an invasive species often do not result in colonization by desirable native species (Kettenring and Reinhardt Adams 2011; Suding 2011). Changes are temporary and do not necessarily lead to habitat improvement. We advocate increased research into the outcomes of *Phragmites* management, the efficacy of management strategies and preplanning to assess which sites to manage (i.e. tradeoffs between management efforts and potential gains). Research can be used to guide landscape-scale multi-year removals that are structured to allow monitoring and adaptive responses to address challenges and meet management outcomes. Programmes should also consider possible underlying causes for *Phragmites* invasion (shoreline buffers to prevent disturbance from development and excess nutrient inputs) and broadening partnerships between ecologists, managers and policy makers (*sensu* Suding 2011) to manage *Phragmites* in a more holistic manner.

## Sources of Funding

This manuscript is the direct outcome of the ‘*Phragmites australis* in North America and Europe’ symposium at the 2011 meeting of the Society of Wetland Scientists and was sponsored by *AoB PLANTS*. E.L.G.H., K.M.K. and D.F.W. are partially funded by NOAA (grant #NA09NOS4780214). Funding support for T.J.M. was provided from MD Sea Grant Award SA7528114-WW and NSF DEB-0950080. E.L.G.H. is supported by the Utah State University Ecology Center, Delta Waterfowl, and a Smithsonian Institution Predoctoral Fellowship. D.M.B. was supported by the Class of 1937 Professorship in Marine Science; Jackson Estuarine Laboratory Contribution #514. This is publication #14-001 of the NOAA/CSCOR Mid-Atlantic Shorelines project.

## Contributions by the Authors

E.L.G.H. and T.J.M. are equal contributors in structuring and researching the manuscript. Other authors contributed equally to their respective expertise.

## Conflicts of Interest Statement

None declared.
